# NF-κB suppresses apoptosis and promotes bladder cancer cell proliferation by upregulating survivin expression *in vitro* and *in vivo*

**DOI:** 10.1038/srep40723

**Published:** 2017-01-31

**Authors:** Xiaolu Cui, Dezhi Shen, Chuize Kong, Zhe Zhang, Yu Zeng, Xuyong Lin, Xiankui Liu

**Affiliations:** 1 Department of Urology, The First Hospital of China Medical University, Shenyang 110001, Liaoning, China; 2 Department of Pathology, The First Affiliated Hospital and College of Basic Medical Sciences, China Medical University, Shenyang 110001, Liaoning, China

## Abstract

Nuclear factor kappa-B (NF-κB) activation is a common phenomenon in cancers, which results in the aberrant expression of NF-κB target genes and leads to malignant transformation, metastatic dissemination, abnormal cell proliferation or resistance to cell death. Survivin is a unique member of the IAP family, a well-known cancer-specific molecule and a molecular marker of poor clinical outcome in several cancer types, including bladder cancer. YM-155, a potent survivin suppressor, has been shown to have anti-tumor activity in preclinical cell lines, xenograft models and phase I/II studies. In the present study, we investigated the function of the NF-κB/survivin pathway in bladder cancer. We found that NF-κB can promote cell cycle progression and reduce apoptosis by upregulating survivin expression, thereby increasing cellular proliferation. We further confirmed the tumorigenic function of the NF-κB/survivin pathway *in vivo* using a xenograft tumor model of stable NF-κB-overexpressing 5637 cells. Moreover, we found that YM-155 significantly induced apoptosis and decreased cellular proliferation as well as tumor growth in mice. Our results demonstrate the carcinogenic function of the NF-κB/survivin pathway in bladder cancer and the role of YM-155 as a promising agent for the strategic treatment of bladder cancer.

Bladder cancer (bladder urothelial carcinoma) is one of the most common cancers and is a predominant genitourinary malignancy and health issue worldwide^1^,^2^,^3^. Existing alternative management strategies, such as chemotherapy and radical cystectomy, have seldom elicited satisfactory effects on the recurrence or metastases of bladder cancer^4^,^5^. Despite continuous advances in surgical techniques, perioperative chemotherapy and radiotherapy, the overall 10-year survival after radical cystectomy remains grave^1^,^3^. Therefore, the mechanism underlying the tumorigenesis and progression of bladder cancer urgently requires investigation for the development of novel therapeutic agents^6^.

The induction of apoptosis and the inhibition of proliferation are crucial aspects of anti-cancer therapies^7^. Alterations in apoptosis can lead to carcinogenesis (e.g., neoplastic cells that live longer and develop resistance to stress). Endogenous cell death pathways are likely to play an important role against malignant transformation^8^. Survivin, encoded by the baculoviral inhibitor of apoptosis repeat-containing 5 (BIRC5) gene, is a unique inhibitor of apoptosis^9^. It is a member of the inhibitor of apoptosis protein (IAP) family, contains 142 amino acids, and is located on chromosome 17 (17q25)^10^,^11^. It is mainly expressed in embryonic tissues and in the majority of tumors but is not in normal differentiated cells^12^,^13^,^14^. The major function of survivin is mitotic progression regulation and apoptosis inhibition^9^,^15^. This multifunctional protein regulates cell division at the G2/M phase and suppresses apoptosis by inhibiting caspase activities^11^,^16^. Elevated expression of survivin in tumors is associated with an advanced cancer stage, poor prognosis and reduced responsiveness to chemotherapy^17^,^18^,^19^. Thus, survivin is a suitable target for cancer therapy. Moreover, survivin has been demonstrated to be a strong, independent predictor of bladder cancer recurrence and cancer-specific survival^17^,^20^,^21^. Therefore, an in-depth study targeting the survivin regulation network is being pursued for novel anti-cancer therapeutics.

Many studies have focused on the role of IAPs as modulators of nuclear factor kappa-B (NF-κB), which has been proposed to be a crucial regulatory family for inflammation, immunity and cell survival and is hallmark of tumorigenesis^10^,^22^. For example, it has been established that c-IAP1 and c-IAP2 form a complex with TNF receptor 1 (TNFR1) and promote TNFα-induced NF-κB activation^23^,^24^. Moreover, the assembly of a survivin-XIAP complex also functions as an effective activator of NF-κB^25^. On the other hand, the importance of the regulatory function of NF-κB in cancer is in the transcription of growth-promoting and anti-apoptotic genes. As reported earlier, in some cancer types, NF-κB inhibits apoptosis by targeting BCL2 and/or IAPs^26^,^27^,^28^. Nonetheless, the precise mechanisms of NF-κB activation and its regulatory role in cell survival and anti-apoptosis in bladder cancer remain unclear.

In this study, we investigated the mechanisms of cell survival in bladder cancer cells. We confirmed that NF-κB activation contributes to the upregulation of the survivin gene in bladder cancer, and we revealed that by upregulating survivin expression, NF-κB enhances the proliferation and suppresses the apoptosis of bladder cancer cell lines both *in vitro* and *in vivo*. In addition, YM-155, a selective inhibitor of survivin, efficiently inhibited survivin expression and xenograft tumor growth.

## Results

### Expression of NF-κB and survivin is upregulated in bladder cancer cell lines and tissue specimens, and a positive correlation is detected in tumor tissue specimens

**Figure 1:**
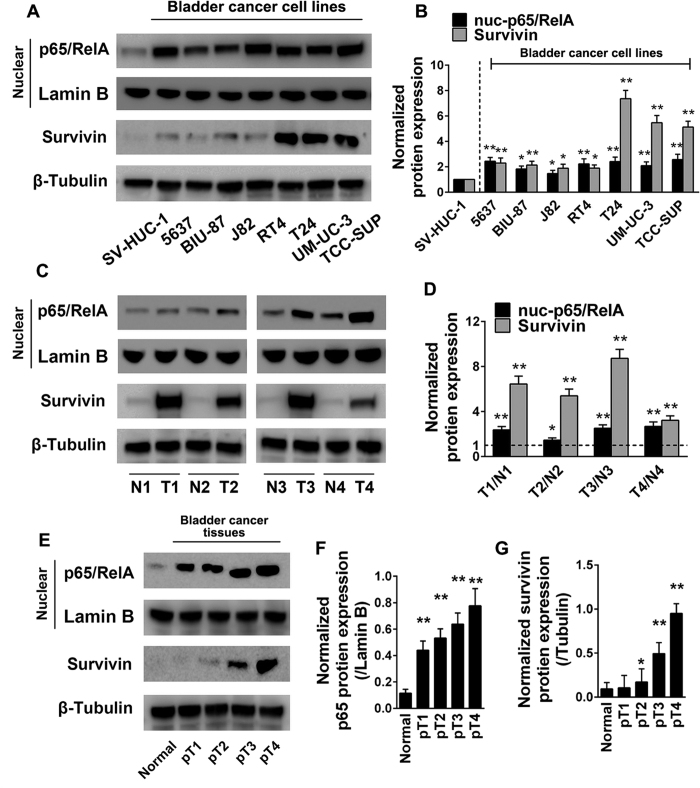
Expression of NF-κB RelA/p65 and survivin is upregulated in bladder cancer cell lines and tissues. (**A,B**) Protein expression of survivin and NF-κB RelA/p65 was detected in one immortalized ureter urothelial cell line and seven bladder cancer cell lines. (**C,D**) Survivin and RelA/p65 expression was compared in four pairs of bladder cancer tissue and adjacent normal bladder mucosa tissue samples by western blotting. (**E,F**) One normal bladder mucosa tissue and four bladder cancer tissues which were pathologically diagnosed with bladder urothelial carcinoma staging from pT1 to pT4, were examined by western blotting analysis. The gels were run under the same experimental conditions. The band intensities were calculated by AlphaEase FC software. β-Tubulin was used as an internal control for total protein measurement, and lamin B1 was used as a nucleoprotein reference. The ratio of target gene to β-Tubulin/lamin B1 was used to conduct the statistical analysis. *P < 0.05 and **P < 0.01, as determined by Student’s T-test.

**Figure 2:**
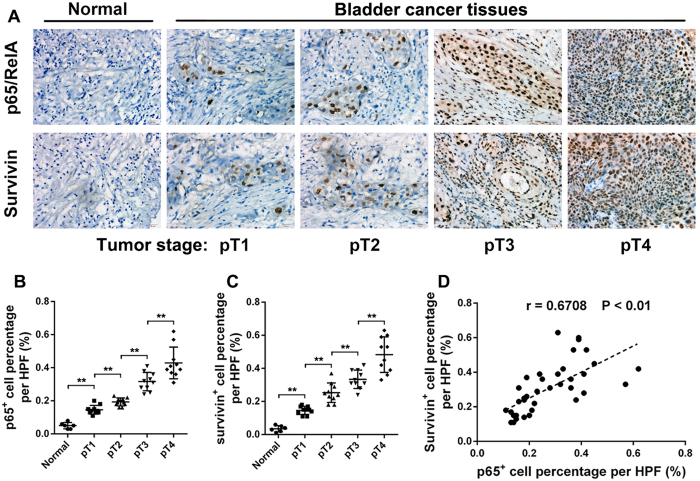
Expression of p65/RelA and survivin is positively correlated in clinical tumor tissue specimens, and correlated with the progression of bladder cancer. (**A**) Immunohistochemistry staining of survivin and p65/RelA in clinical tissue specimens. Figures are visualizations of five representative cases. The survivin/p65 positive expression cells were counted in three randomly observed visual field (magnification of 400X) and the survivin/p65 positive cell proportion was used to conduct the statistical analysis. (**B,C**) Expression of survivin and p65/RelA is significantly correlated with clinical tumor progression. (**D**) Pearson’s correlation coefficient analysis revealed a significant positive correlation between expression of survivin and p65/RelA in forty bladder cancer tissue specimens (R = 0.6708, P < 0.01). **P < 0.01, as determined by Student’s T-test.

We first investigated the survivin and NF-κB subunit p65/RelA expression profiles in one immortalized ureter urothelial cell line and seven bladder cancer cell lines by western blotting. As shown in Fig. 1A,B, bladder cancer cell lines exhibited significantly increased survivin and nuclear p65/RelA expression compared to the ureter urothelial cell line. Next, we detected the expression of the above two proteins in four pairs of bladder cancer and adjacent normal tissue specimens. Bladder cancer tissue specimens revealed significant upregulated survivin and nuclear RelA expression compared with that of adjacent normal mucosa tissue specimens (Fig. 1C,D). Then, we detected survivin and p65/RelA expression in one normal bladder mucosa tissue and 4 bladder cancer tissues which were diagnosed with bladder urothelial carcinoma staging from pT1 to pT4 by western blot. The results suggested that with the pathological progression of bladder cancer, expression of survivin and p65/RelA revealed significant elevation (Fig. 1E–G). To further confirm the expression pattern and correlation of these two proteins in clinical tissue specimens, immunostaining analysis (Fig. 2A) was performed in 5 normal bladder mucosa tissue specimens and 40 bladder cancer tissue specimens which were diagnosed with bladder urothelial carcinoma staging from pT1 to pT4 (10 cases/stage). The results (Fig. 2B,C) showed that with the pathological progression of bladder cancer, survivin and p65/RelA expression significantly increased. Furthermore, Pearson’s correlation coefficient analysis (Fig. 2D) suggested a significant positive correlation between expression of survivin and p65/RelA in 40 bladder cancer tissue specimens (R = 0.6708, p < 0.01). We also assessed the association of the survivin and p65/RelA expression level with the clinical and pathological characteristics of these patients. As shown in Table 1, survivin and NF-κB p65 were highly expressed in high-grade tumors, tumors with metastases and Lymphatic invasion. These results suggest that the expression of survivin and NF-κB p65/RelA is not only remarkably upregulated in bladder cancer cell lines and tissues but also closely correlated with the clinical progression of bladder cancer, moreover, expression of the two proteins is positively correlated in bladder cancer tissue specimens.

### NF-κB activation enhances survivin expression in bladder cancer cell lines

**Figure 3:**
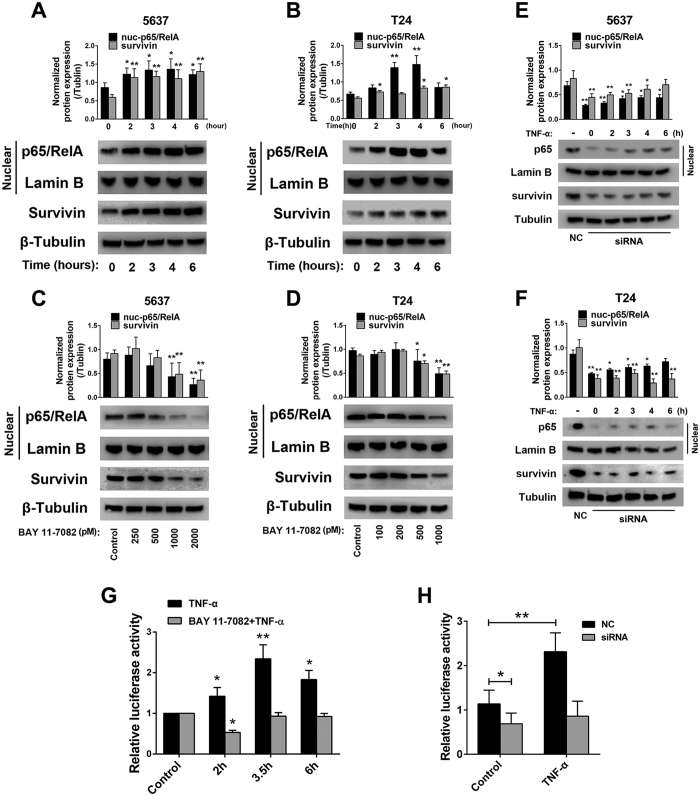
NF-κB activation enhances survivin expression by binding with the promoter in bladder cancer cell lines. (**A,B**) 5637 and T24 cell lines were treated with TNF-α (50 ng/ml) for 2, 3, 4, and 6 h. Survivin and NF-κB expression was detected by western blotting. (**C,D**) The tested cells were treated with increasing doses of BAY 11-7082 for 24 h, and the expression of survivin and NF-κB was detected by western blotting. The expression of survivin was consequently increased or decreased with the activation or deactivation of NF-κB activity, respectively. (**E,F**) The tested cells were transfected with NC/p65 siRNA, 24 hours post-transfection cell were treated with TNF-α (50 ng/ml) for 2, 3, 4, and 6 h. (**G**) The activation of NF-κB signaling significantly enhanced survivin promoter luciferase activity. In contrast, pretreatment with BAY 11-7082 (2 μM for 2 h) effectively hampered the increase in luciferase activity induced by TNF-α (50 ng/ml). (**H**) p65 knockdown significantly decreased the luciferase activity which was otherwise stimulated by TNF-α. The gels were run under the same experimental conditions. The band intensities were calculated by AlphaEase FC software. β-Tubulin was used as an internal control for total protein measurement, and lamin B1 was used as a nucleoprotein reference. The ratio of target gene to β-Tubulin/lamin B1 was used to conduct the statistical analysis. *P < 0.05 and **P < 0.01, as determined by Student’s T-test.

We next asked whether NF-κB activation contributes to the upregulation of survivin expression in bladder cancer. To test this hypothesis, we successively activated or deactivated NF-κB signaling using tumor necrosis factor-α (TNF-α) or BAY 11–7082, respectively. TNF-α activates the classical NF-κB signaling pathway, whereas BAY 11–7082 selectively inhibits the phosphorylation of IκB kinase (IKK) and leads to the deactivation of NF-κB signaling^29^. First, we treated the 5637 and T24 bladder cancer cell lines with TNF-α (50 ng/ml) for 0, 2, 3, 4 and 6 h. Next, we measured survivin and nuclear p65/RelA protein expression by western blotting analysis. As shown in Fig. 3A,B, NF-κB was activated by TNF-α in a time-dependent manner, and survivin expression was relatively upregulated. Next, we treated the tested cells with BAY 11–7082 using increasing concentrations of 0.25, 0.5, 1 and 2 μM for the 5637 cells and 0.1, 0.2, 0.5 and 1 μM for the T24 cells (Fig. 3C,D). Our results suggest that survivin expression exhibits significant downregulation, which is a general reflection of the level of inhibition of NF-κB signaling. Next, to avoid the influence that TNF-α or BAY 11–7082 may cause on survivin expression through other pathways rather than NF-κB, we knocked down p65/RelA with siRNA oligo, then the cells were treated with TNF-α in the same method. Gene knockdwon efficiency was measured by western blot and real-time quantitative PCR analysis (Figure SB). Survivin and p65 protein expression was detected in different time points after TNF-α treatment by western blot. Figure 3E,F suggested that p65/RelA expression was remarkably inhibited by siRNA transfection, accordingly, survivin expression revealed a relative downregulation, furthermore, there was no significant upregulation of the two proteins after TNF-α stimulation. Taken together, these results suggest that the activation of the NF-κB signaling pathway significantly contributes to the upregulation of survivin expression, whereas deactivation of NF-κB downregulates survivin expression.

### NF-κB overexpression stimulates the luciferase activity of the survivin promoter

Multiple transcription enhancer-binding sites within the 5′ flanking region of the survivin gene have been identified, including four NF-κB sites^30^,^31^. In B-cell lymphomas^26^, colorectal cancer^27^ and T-cell leukemia^31^, NF-κB has been reported to bind to the promoter region of the survivin gene and enhance transcription. Hence, we further validated whether NF-κB induces survivin expression by binding to the promoter region in bladder cancer cells. A luciferase reporter plasmid containing the survivin promoter region was constructed (Figure SA), and a dual luciferase reporter assay was performed to measure the interaction. After transfection with the luciferase plasmid, the cells were treated with TNF-α (50 ng/ml) alone or pretreated with BAY 11–7082 (2 μM) for 2 h followed by the same TNF-α treatment. As shown in Fig. 3G, TNF-α significantly stimulated the luciferase activity of the survivin promoter luciferase construct, whereas BAY 11–7082 pretreatment significantly inhibited the luciferase activity, which was otherwise stimulated by TNF-α alone. Furthermore, we transfected the cells with negative control (NC) oligo or siRNA against p65, after 24 hours the cells were separately treated with control/TNF-α, and the cell luciferase activity was measured after another 24 hours. Figure 3H showed that TNF-α remarkably stimulated the luciferase activity of the cells received transfection of NCs, in contrast, survivin promoter luciferase activity of p65 knockdown cells could no longer be upregulated by TNF-α treatment.

### YM-155 significantly decreases survivin expression and inhibits bladder cancer cell proliferation

Next, we investigated the carcinogenic function of the NF-κB/survivin pathway in bladder cancer cells. The imidazolium-based agent Sepantronium bromide (YM-155) was used to inhibit survivin expression. YM-155 has been confirmed to specifically decrease survivin expression and to induce apoptosis in many cancers^32^,^33^. Moreover, recent phase II studies have revealed encouraging anti-tumor activities of YM-155 combined with other anti-neoplastic agents^34^,^35^. However, few studies have reported the effects of YM-155 in bladder cancer. Therefore, it is necessary to determine its function in bladder cancer cell lines.

**Figure 4:**
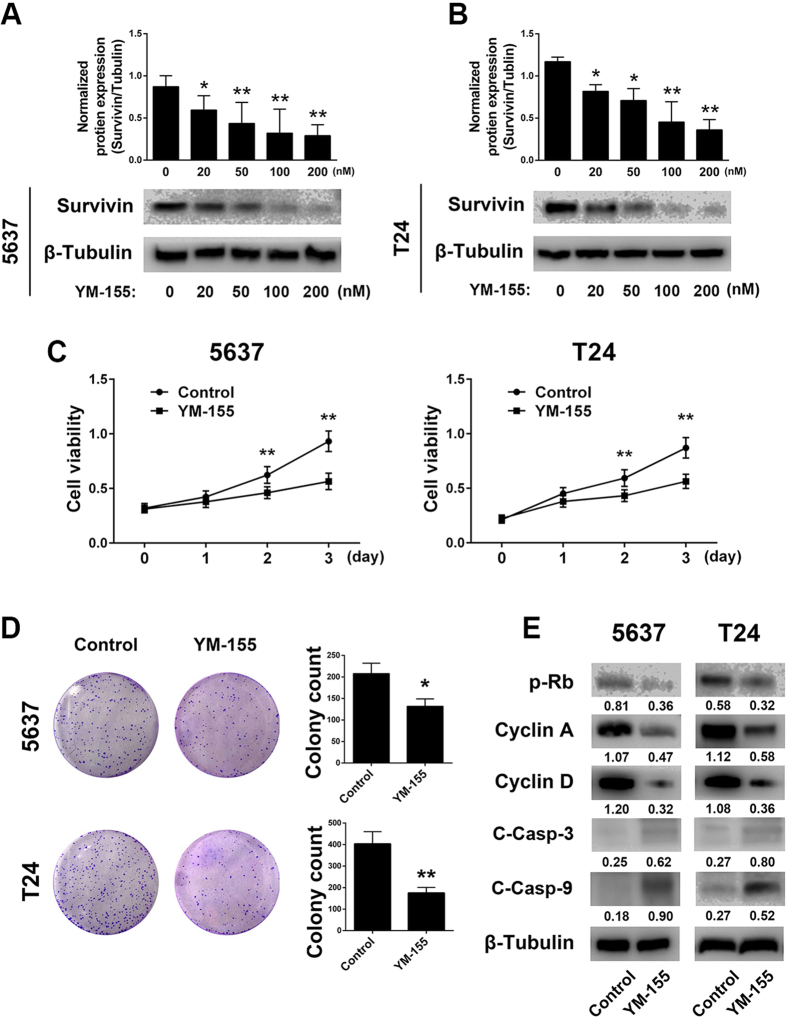
YM-155 significantly decreases survivin expression and inhibits bladder cancer cell proliferation. (**A**) 5637 and T24 cells were treated with increasing doses of YM-155 for 24 h, and then survivin expression was detected by western blotting. (**B,C**) CCK8 cell proliferation analysis and cell colony formation assay revealed that YM-155 significantly decreased the proliferation of bladder cancer cell lines. Moreover, the cell survival- and apoptosis-related proteins p-Rb, cyclin A, cyclin D, cleaved caspase-3 and cleaved caspase-9 were detected by western blotting. (**D**) YM-155 inhibits cell proliferation and cell cycle arrest by decreasing the expression of p-Rb, cyclin A and cyclin D and inducing cell apoptosis by activating the caspase pathway. The final concentration of YM-155 for cell treatment was 100 nM. The gels were run under the same experimental conditions. The band intensities were calculated by AlphaEase FC software; the ratios of target gene to β-tubulin were used to perform statistical analyses. β-Tubulin was used as the internal control. *P < 0.05 and **P < 0.01, as determined by Student’s T-test.

We treated 5637 and T24 cells with YM-155 and found that the agent significantly decreased survivin expression in a dose-dependent manner (Fig. 4A). Next, we treated the cells with YM-155 (100 μM) for 24 h, and cell proliferation was examined by CCK8 and colony formation assays (Fig. 4B,C). Meanwhile, the cell cycle- and apoptosis-related proteins p-Rb, cyclin A, cyclin D, cleaved-caspase 3 and cleaved-caspase 9 were also detected by western blotting (Fig. 4D). Similar to findings of previous studies, YM-155 specifically inhibited proliferation and induced apoptosis and the cycle arrest of bladder cancer cells.

### NF-κB suppresses apoptosis and increases the proliferation of bladder cancer cells by targeting survivin

**Figure 5:**
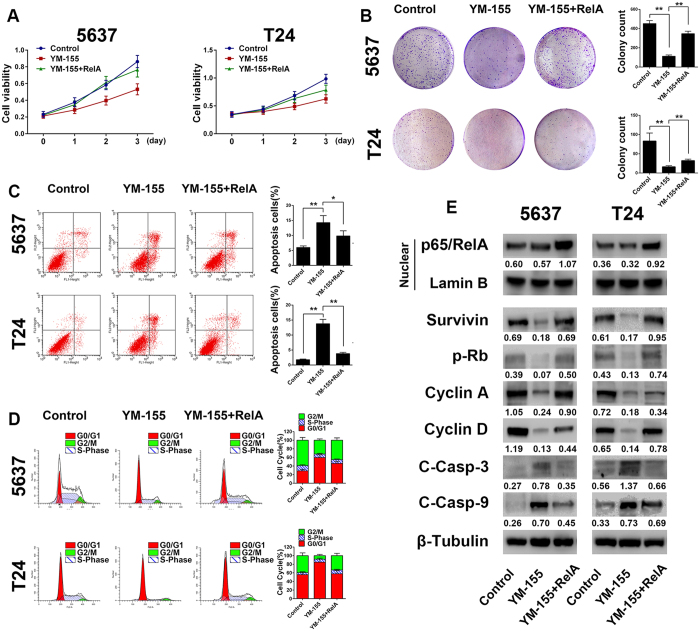
NF-κB increases proliferation and suppresses apoptosis of bladder cancer cells by targeting survivin *in vitro*. (**A**) A CCK8 assay showing that YM-155 significantly inhibits the proliferation of the tested cells. In contrast, the overexpression of NF-κB p65/RelA reverted cell proliferation, which was impaired by YM-155. (**B**) A cell colony formation assay revealed similar results. Next, we further investigated the effect of YM-155 and NF-κB overexpression on cell cycle and apoptosis by flow cytometric analyses. (**C**) Analysis of apoptosis revealed that YM-155 dramatically induced apoptosis in both cell lines, and NF-κB overexpression plus YM-155 treatment significantly inhibited apoptosis compared with YM-155 treatment alone. (**D**) Cell cycle analysis suggests that YM-155 treatment arrested cell cycle progression in the G0/G1 phase. In contrast, G2/M phase populations were significantly increased in the NF-κB overexpression plus YM-155 treatment group compared with the YM-155 treatment alone group. (**E**) The expression profiles of the cell survival- and apoptosis-related proteins described above were detected. YM-155 induced apoptosis by activating the caspase pathway and inhibiting the expression of p-Rb, cyclin A and cyclin D. In contrast, NF-κB overexpression with YM-155 treatment significantly decreased cleaved caspase 3/9 expression and reverted the expression of p-Rb, cyclin A and cyclin D compared with YM-155 treatment alone. The final concentration of YM-155 for the treatment of cells was 100 nM. The gels were run under the same experimental conditions. The band intensities were calculated by AlphaEase FC software. β-Tubulin was used as an internal control for total protein measurement, and lamin B1 was used as a nucleoprotein reference. The ratio of target gene to β-Tubulin/lamin B1 was used to conduct the statistical analysis. *P < 0.05 and **P < 0.01, as determined by Student’s T-test.

NF-κB activation is a common phenomenon in cancers, which results in the aberrant expression of distinct sets of NF-κB target genes, leading to malignant transformation, metastatic dissemination, abnormal cell proliferation or resistance to cell death^36^. In this context, we investigated whether NF-κB promotes cell survival by upregulating survivin expression in bladder cancer. Apoptosis was induced by YM-155 treatment. Next, the p65/RelA expression plasmid was transfected to activate NF-κB. CCK8 and colony formation assays (Fig. 5A,B) revealed that YM-155 remarkably reduced the proliferation of the tested cells, whereas NF-κB overexpression reverted the impaired cell proliferation, which was otherwise inhibited by YM-155 alone. Flow cytometric analysis further demonstrated the alterations in the cell cycle and apoptosis. As shown in Fig. 5C, YM-155 alone significantly induced apoptosis, and NF-κB overexpression combined with YM-155 reduced apoptosis compared with YM-155 treatment alone. Similarly, a cell cycle analysis (Fig. 5D) suggested that YM-155 alone significantly impaired cell cycle progression at the G0/G1 phase, whereas NF-κB overexpression combined with YM-155 treatment promoted cell cycle progression to the G2/M phase compared with YM-155 treatment alone. Next, we measured survivin and p65/RelA expression as well as that of the cell cycle- and apoptosis-related proteins p-Rb, cyclin A, cyclin D, cleaved-caspase 3 and cleaved-caspase 9 within each group by western blotting (Fig. 5E). Our *in vitro* results suggested that YM-155 potently induced apoptosis and inhibited the proliferation of bladder cancer cell lines by downregulating survivin expression. In contrast, the recovery of survivin expression induced by NF-κB overexpression significantly reduced the anti-tumor effects of YM-155. These findings indicate that NF-κB suppresses apoptosis and increases the proliferation of bladder cancer cells by upregulating survivin.

### NF-κB promotes tumor growth by upregulating survivin expression *in vivo*

**Figure 6:**
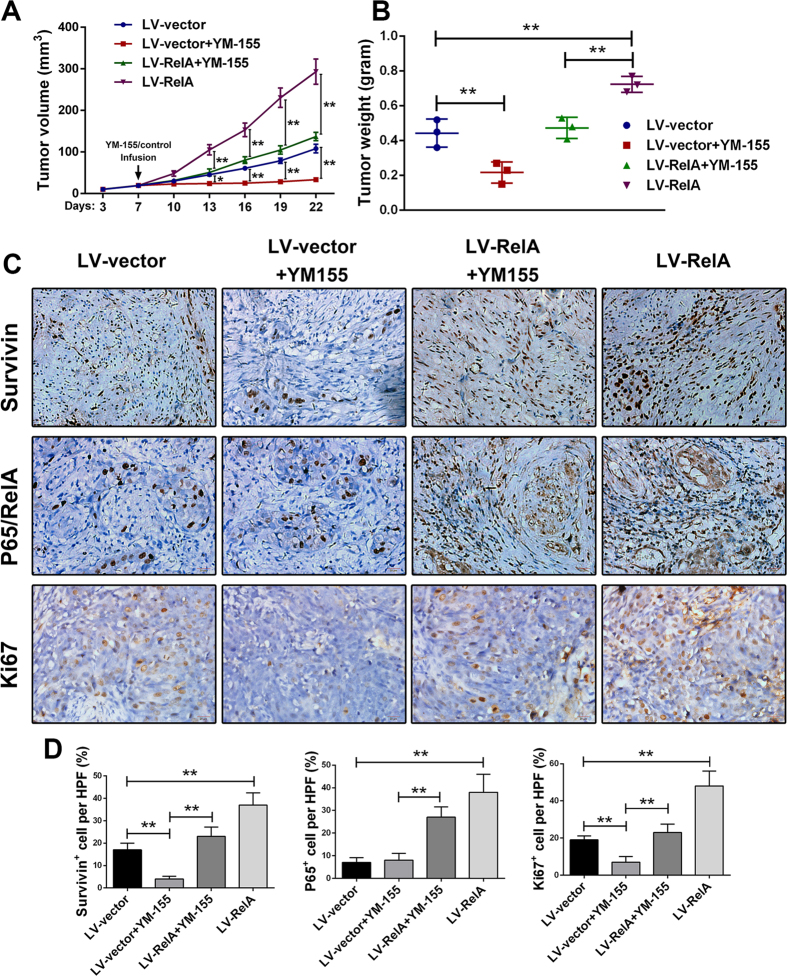
NF-κB promotes tumor growth by upregulating survivin expression *in vivo*. (**A**) Xenograft tumor growth was monitored and analyzed. Tumor volumes were measured on the indicated days and are presented as the mean values ± SD. (**B**) Tumor weights were also measured and analyzed. (**C,D**) The tumors were immunostained using survivin, NF-κB p65/RelA and ki67 antibodies. The expression of survivin, p65/RelA and ki67 in each group was measured and analyzed by counting the positive cell percentages in three random visual fields. Original magnification: 400x. *P < 0.05 and **P < 0.01, as determined by Student’s T-test.

We next investigated the carcinogenic function of the NF-κB/survivin pathway in bladder cancer using a xenograft mouse model. For NF-κB stable overexpression, we used recombinant lentiviruses encoding the CDS sequence of p65/RelA (purchased from GenePharma, Shanghai, China) to infect 5637 cells; a negative control lentiviral vector encoding a nonsense sequence was used as a vehicle control. The stably transduced 5637 cells were selected using puromycin. To assess the efficiency of lentiviral gene delivery, p65/RelA expression was assessed by western blotting at 20 days post-infection (Figure SC>). Lv-NC-5637 and Lv-RelA-5637 cells were separately injected into the flanks of athymic nude mice to establish xenograft tumors. When tumor sizes reached approximately 20 mm^3^, the mice were divided into four groups (2 groups received Lv-NC-5637 injection, and 2 groups received Lv-RelA-5637 injection; 3 mice/group). The groups of mice with tumors established by Lv-NC-5637 or Lv-RelA-5637 were continuously infused with YM-155 at a dose of 5 mg/kg/day for 7 days; the other two groups of mice were infused with the vehicle control. Tumor growth was monitored throughout the survival period, and the mice were euthanized and tumors were excised at 15 days after infusion (Figure SE). The tumor growth curve (Fig. 6A) showed that the tumors in the YM-155 plus Lv-NC-5637-treated mice grew significantly slower than those in the vehicle control plus Lv-NC-5637-treated mice. In contrast, in the NF-κB-overexpressing (Lv-RelA-5637) groups, the tumor growth inhibition resulting from YM-155 was significantly reduced, and the tumors in the mice treated with vehicle control plus Lv-RelA-5637 revealed the fastest growth rate among all of the groups. Accordingly, the same was observed in terms of tumor size and weight measurements (Fig. 6A,B).

To further determine whether NF-κB induces survivin expression in xenograft tumors, the tumor tissues were dissected and subjected to IHC staining. Our results (Fig. 6C,D) demonstrated that YM-155 significantly inhibited survivin expression compared with the vehicle control, whereas NF-κB overexpression reverted the survivin expression that was otherwise inhibited by YM-155; NF-κB overexpression without YM-155 treatment significantly upregulated survivin expression. Ki67 expression was also detected, and in accordance with the tumor growth, NF-κB overexpression without YM-155 treatment revealed the highest expression level of ki67, whereas YM-155 with vector significantly decreased ki67 expression and tumor growth. The data detailed above suggest that *in vivo*, NF-κB enhances the proliferation and resistance to apoptosis of bladder cancer cell lines by upregulating survivin expression.

## Discussion

In this study, we have shown that survivin serves as a downstream mediator of NF-κB signaling in bladder cancer malignant progression. We have provided clear evidence that in bladder cancer, NF-κB activation enhances the expression of survivin both *in vitro* and *in vivo*. Accordingly, with survivin overexpression, cell proliferation and resistance to apoptosis is significantly stimulated, which further leads to increased xenograft tumor growth. Moreover, the YM-155 agent potently suppresses the expression of survivin in bladder cancer cells, inducing apoptosis and cell cycle arrest, consequently suppressing xenograft tumor growth. Our study further demonstrates that the NF-κB/survivin pathway plays a tumorigenic function in bladder cancer cell lines, and for the first time, we document the mechanism of YM-155 in bladder cancer tumor growth in a nude mouse model.

The major issues underlying poor clinical outcome and even metastasis-related death in bladder cancer patients have been identified in tumors prone to progression, recurrence and chemoradiation resistance^37^,^38^. To overcome these issues, a number of molecular markers have been studied and are suggested to be involved in the biological process of malignant transformation of bladder cells (e.g., the cell cycle regulators p53, pRb and cyclin A, as well as the apoptosis mediators IAP and Bcl-2 family proteins^19^,^38^,^39^,^40^). NF-κB is a family of nuclear transcription regulators, which affect cellular responses to multiple extracellular stimuli^41^. Moreover, constitutive NF-κB activation, which commonly occurs in malignant tissues, results in the strong transactivation of a wide variety of reporter genes, resulting in perturbations in cell cycle progression, resistance to apoptosis and enhanced angiogenesis^36^. Several studies have demonstrated that NF-κB activation, as indicated by nuclear p65/RelA subunit expression, is positively associated with tumor histological grade, T-category and chemoradiation resistance, particularly in muscle-invasive bladder cancers^42^,^43^. In this study, we detected a significant increase in nuclear p65/RelA levels in bladder cancer tissues compared to that of adjacent normal tissues, and p65/RelA expression was also correlated with pathological progression of bladder cancer. Moreover, our xenograft tumor model revealed increased tumor growth in the mice receiving injections of stable NF-κB-overexpressing 5637 cells. These results are consistent with previous studies.

The inhibitor of apoptosis (IAP) protein family, which was originally defined as a class of endogenous caspase inhibitors, has garnered increasing attention for its disparate biological functions in survival, mitosis and intracellular signaling^11^. The Baculovirus IAP Repeat (BIR) domain is the defining structural characteristic of IAP molecules, which mediate protein recognition and protein-to-protein interactions^10^. Several IAPs also contain additional structural domains, such as the carboxyl-terminus RING, an ubiquitin-associated domain, that facilitates the ubiquitination process^10^. Survivin is a unique member of the IAP family, a well-known cancer-specific molecule, and a molecular marker for poor clinical outcome of tumors, including bladder cancer^44^,^45^. Indeed, it has been widely reported that survivin is a target of NF-κB signaling and promotes cancer progression as well as drug resistance in many cancers. However, to date, no reports have been made to confirm the NF-κB/survivin pathway in bladder cancer using a stable NF-κB-overexpressing xenograft tumor model. Here, using a xenograft tumor assay, we not only demonstrated the NF-κB/survivin axis both *in vitro* and *in vivo*, but we also tested the anti-cancer function of YM-155.

YM-155, a small molecule that selectively suppresses survivin mRNA expression, has been investigated in preclinical cell lines, xenograft models and phase I/II studies^33^,^34^,^35^,^46^. YM-155 has been shown to be safe and well tolerated at an ideal dose of approximately 5 mg/kg/day by continuous infusion for 7 days, according to phase I and II studies^47^,^48^. In a study concerning the anti-tumor activities of YM-155 in a wide variety of human cancer cell lines and xenograft models, YM-155 was reported to elicit significant anti-tumor activity in a bladder cancer (UM-UC-3) xenograft model^49^. In our study, we inoculated nude mice with the bladder cancer cell line 5637, and YM-155 or vehicle control was continuously administered at 5 mg/kg/day for 7 days. Our results showed that YM-155 potently suppresses xenograft tumor growth, as well as the expression of the survivin gene. Importantly, it also significantly hampered the tumor growth induced by NF-κB activation.

In bladder cancer, survivin mediates resistance to apoptosis and cell proliferation induced by NF-κB both *in vitro* and *in vivo*. Finally, YM-155 is a promising agent for bladder cancer management.

## Methods

### Cell culture

The human bladder urothelial carcinoma cell lines (T24, 5637, J82, BIU, RT4, UM-UC-3, and SW-780) and immortalized human ureter urothelial cell line (SV-HUC-1) were cultured in RPMI-1640 medium (HyClone, Logan, UT, USA) supplemented with 10% FBS (HyClone) and 1% penicillin-streptomycin (HyClone) at 37 °C under a humidified atmosphere with 5% CO_2_. For NF-κB activation, recombinant human TNF-α (R&D systems, Minneapolis, MN, USA) was added to the cells (diluted in serum-free medium to a final concentration of 50 ng/ml). For the control group, the cells were cultured in serum-free medium without TNF-α. For NF-κB inhibition, BAY 11–7082 (Selleckchem, Houston, TX, USA) was added to the cells at the indicated concentrations, and 10 μl of DMSO was added per 1.0 ml of medium as a control. YM-155 (Selleckchem, Houston, TX, USA) was diluted in DMSO at a storage concentration of 1 mM and added to the cells at the indicated concentrations; 10 μl of DMSO was added per 1.0 ml of medium as a control.

### RNA extraction and real-time quantitative PCR

Total RNA was extracted from cultured cell lines using TRIzol reagent (Invitrogen, Carlsbad, CA, USA) and reverse transcribed with random primers using PrimeScript™ RT Master Mix (Takara Biotechnology, Dalian, Liaoning, China) according to the manufacturer’s instructions. qRT-PCR was performed to detect the levels of PKCs and β-actin using SYBR Premix Ex Taq™ (Takara Biotechnology, Dalian, Liaoning, China) and LightCycler^TM^ 480 II system (Roche, Basel, Switzerland). The β-actin was used as the internal control for each gene. The primer sequences were as following:


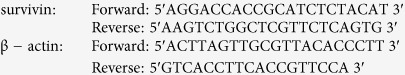


The relative levels of expression were quantified and analyzed using LightCycler^TM^ 480 software 1.5.1.6.2 (Roche, Basel, Switzerland). The real-time value for each sample was averaged and compared using the Ct method. The relative expression level (defined as a fold change) of each target gene (2^−ΔΔCt^) was normalized to the endogenous β-actin reference (ΔCt) and related to the amount of target gene in the control sample, which was defined as the calibrator at 1.0. Three independent experiments were performed to analyze the relative gene expression, and each sample was tested in triplicate.

### Patients and tissue specimens

Four pairs of bladder urothelial carcinoma tissues and adjacent normal mucosa tissues (located > 3 cm from the tumor) were freshly collected from four patients. Another one non-tumor bladder mucosa tissue specimen and four bladder urothelial carcinoma tissue specimens were freshly collected from the other four patients who were pathologically diagnosed with bladder urothelial carcinoma staging from pT1 to pT4. The patients were hospitalized, pathologically diagnosed with bladder urothelial carcinoma and underwent transurethral bladder tumor resection or radical cystectomy at the Urology Department at the First Hospital of China Medical University (Shenyang, China). The study was conducted according to an institutional review board-approved protocol (2012–33) by Medical Ethics Committee of the First Affiliated Hospital of China Medical University, and written informed consent was obtained from each patient for surgery and research purposes. All of the cases were classified according to the 1997 UICC TNM classification for the stage and according to OMS 2004 for the grade. The pathological sections of five non-tumor bladder mucosa tissue specimens and forty bladder cancer tissue specimens were provided by Department of Pathology at the First hospital of China Medical University, and the pathological diagnosis and analysis of immunochemistry staining result in this study were made in collaboration with Department of Pathology.

### Small interfering RNA, plasmid construction and retroviral infection

Three pairs of siRNAs against p65/RelA were synthesized by GenePharm (GenePharma Corporation, Shanghai, China). The Sequences of siRNAs were as following:


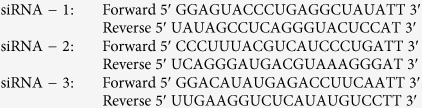


The pGL3-survivin-promoter luciferase plasmid was purchased from GenePharm (GenePharma Corporation, Shanghai, China). The recombinant lentivirus vector pGLV-NF-κB and the negative control vector (pGLV-NC) containing a nonsense sequence were purchased from GenePharm (GenePharma Corporation, Shanghai, China). We transfected 5637 cells with pGLV-NF-κB or pGLV-NC for 3 days. Then, the stably transduced 5637 cells were selected using puromycin for 20 days by adding the minimum concentration of puromycin required to kill untransduced 5637 cells. The efficiency of retroviral infection was measured by western blotting.

### Cell proliferation assay

Cell proliferation was determined using Cell Counting Kit-8 (CCK-8) (Dojindo, Tokyo, Japan) and a cell colony formation assay, according to the manufacturer’s protocol. Cells in the logarithmic phase of growth were seeded into 96-well culture plates at 3 × 10^3^ cells per well for 24 h. Next, the cells were differentially treated with TNF-α, BAY 11–7082 or YM-155. At the indicated times, the assays were performed by directly adding the CCK-8 reagent into the medium and incubating in 37 °C for 1 h. The absorbance value was measured at 450 nm to determine cell viability using a 96-well plate reader. (model 680, Bio-Rad, Hertfordshire, UK).

For cell colony formation assay, the cells were plated in 24-well plates (300 cells per well) and incubated for 7 days in complete medium. Colonies were fixed with 10% formaldehyde for 10 min and stained with 1.0% crystal violet for 5 min. The number of colonies, defined as >50 cells/colony, was counted.

### Protein extraction and western blotting

Cells were harvested in RIPA lysis buffer (Beyotime, Shanghai, China) and boiled for 10 min at 90 °C. Protein concentrations were measured using the BCA assay. Approximately, 50 μg of protein extract from cultured cells or 100 μg from fresh surgical bladder tissues were separated by 10% SDS-polyacrylamide gel electrophoresis (SDS-PAGE). The gels were then electrotransferred onto polyvinylidene difluoride (PVDF) membranes (Millipore, Billerica, MA, USA), which were then incubated with the indicated primary antibodies in 5% nonfat milk in TBS-T overnight at 4 °C. Next, the membranes were washed for 15 min each and immediately incubated with anti-rabbit or anti-mouse horseradish peroxidase-conjugated secondary antibodies for 1 h at 37 °C. The immunobands were visualized using ECL detection reagents (Transgen Biotechnology, Beijing, China) on a MicroChemi^TM^ Chemiluminescent Imaging System (DNR Bio-Imaging Systems, Mahale HaHamisha, Jerusalem, Israel). Antibodies against p65/RelA and survivin were purchased from Cell Signaling Technology (Danvers, MA, USA). Antibodies against Cyclin A, Cyclin D, p-Rb, cleaved caspase-3 and cleaved caspase-9 were purchased from Abcam (Cambridge, MA, USA). The housekeeping protein β-Tubulin (Sigma-Aldrich, St. Louis, MO, USA) was used as an internal control for total protein measurement, and lamin B1 (Sigma-Aldrich, St. Louis, MO, USA) was used as a nucleoprotein reference. The densitometric values were calculated by AlphaEase^TM^ FC 6.0 software (Alpha Innotech, Santa Clara, CA, USA), and the ratios of target protein to β-tubulin were used to conduct the statistical analysis.

### Nuclear/cytoplasmic fractionation

A Nuclear and Cytoplasmic Protein Extraction Kit (Beyotime, Shanghai, China) was used to extract the nuclear and cytoplasmic proteins from cultured cells and tissues, according to the manufacturer’s protocol. Briefly, cells were washed with cold PBS, resuspended in buffer containing 1 mM DTT and 1 mM PMSF, and incubated on ice for 15 min. Detergent was added, and the cells were vortexed for 30 s at the highest speed. The nuclei and supernatant (cytoplasm) were separated by centrifugation at 4 °C. The nuclei were resuspended in buffer containing 1 mM DTT and 1 mM PMSF, incubated on ice for 30 min, and vortexed with interruptions. Nuclear extracts were collected by centrifugation at 14,000 × *g* for 10 min at 4 °C. For nuclear protein extraction of tissues, 60 mg of frozen bladder tissues were excised, immediately suspended in buffer containing 1 mM DTT and 1 mM PMSF, homogenized on ice, and then incubated for 15 min. The subsequent procedure was the same as that for cell nuclear and cytoplasmic protein extraction.

### Cell apoptosis and cycle analysis by flow cytometry

Cells (3 × 10^4^ per well) were seeded into 24-well culture plates and cultured for 24 h. Then, the cells were treated with the indicated reagents and methods for the indicated study purpose. The cells were harvested, washed three times in phosphate buffered saline (PBS), and resuspended in 0.4 ml of ice-cold PBS. The resuspended cells were incubated with propidium iodide (PI) and a fluorescein isothiocyanate (FITC)-conjugated monoclonal antibody specific for Annexin V (BD Biosciences, San Diego, CA, USA) for cell apoptosis analysis or PI/RNase staining buffer (BD Biosciences, San Diego, CA, USA) at 4 °C for 30 min for cell cycle analysis. Flow cytometry data were acquired on a Becton Dickinson FACS Calibur (BD Biosciences, San Jose, CA), and the data was analyzed by the ModFit LT software package (BD Biosciences, San Jose, CA). The experiments were performed independently in triplicate for each cell line.

### Dual luciferase reporter assay

Cells (3 × 10^4^ cells per well) were plated into 24-well culture plates and allowed to settle. The cells were separately transfected with p65/RelA overexpression plasmid (pCMV4-RelA-GFP) or the empty vector for 24 h. Then, pGL3 luciferase reporter plasmids, containing the promoter sequence of the survivin/BIRC5 gene, were transfected into the cells for another 48 h. Luciferase and Renilla signals were measured using a Dual Luciferase Reporter Assay Kit (Promega, Madison, WI, USA) according to the manufacturer’s protocol.

### Xenograft tumor model

BALB/c nude mice (4–6 weeks old, 14–16 g) were purchased from Beijing Vital River Experimental Animal Technology Co. Ltd. The mice were housed in barrier facilities on a 12-h light/dark cycle. The study was approved by Medical Laboratory Animal Welfare and Ethics Committee of China Medical University and the methods were carried out in accordance with the approved guidelines. Lv-RelA-5637 and Lv-NC-5637 cells were separately injected into the flanks of athymic nude mice to establish a xenograft tumor. When tumor sizes reached approximately 20 mm^3^, the mice were divided into four groups (two groups were established by Lv-RelA-5637 and the other two by Lv-NC-5637; 3 mice/group). Each of the mice with xenograft tumors expressing Lv-RelA-5637 or Lv-NC-5637 were continuously infused with YM-155 at 5 mg/kg/day for 7 days; the other two groups of mice were infused with vehicle control. Tumors were examined every 3 days; the lengths and widths were measured using calipers, and tumor volumes were calculated using the equation (length × width^2^)/2. At 15 days after infusion, the mice were euthanized, and the tumors were excised and weighed. Next, xenograft tumors were lysed in RIPA lysis buffer (Beyotime, Shanghai, China) for western blotting or fixed in 10% (v/v) buffered formalin and paraffin-embedded for IHC staining.

### Immunohistochemistry

The expression of survivin and p65/RelA in tumor tissues was detected using an UltraSensitive^TM^ Streptavidin-Peroxidase (Mouse/Rabbit) IHC kit (Maxin-Bio, Fuzhou, Fujian, China) according to the manufacturer’s instructions. Briefly, sections were dewaxed in xylene and ethanol. Antigen retrieval was performed using a microwave for 10 min at 100 °C. The sections were then incubated with rabbit anti-survivin antibody (1:200) (Cell Signaling Technology, Danvers, MA, USA) and rabbit anti-p65/RelA antibody (1:100) (Abcam, Cambridge, MA, USA)) for 1 h, followed by biotinylated anti-IgG antibody and streptavidin-biotinylated-complex horseradish peroxidase. For both antigens, DAB (Beyotime, Shanghai, China) and hematoxylin (Beyotime, Shanghai, China) were used for nuclear staining.

### Statistical analysis

The data are shown as the mean values ± sd. All statistical analyses were performed using SPSS (Statistical Package for the Social Sciences) 21.0 statistical software (SPSS Inc., Chicago, IL, USA). A two-tailed Student’s t-test was used to assess significant differences between two groups of data in all pertinent experiments. Pearson’s correlation coefficient analysis was used to determine the correlation between gene expressions. A P-value ≤ 0.05 was considered significant.

## Additional Information

**How to cite this article**: Cui, X. *et al*. NF-κB suppresses apoptosis and promotes bladder cancer cell proliferation by upregulating survivin expression *in vitro* and *in vivo. Sci. Rep.* **7**, 40723; doi: 10.1038/srep40723 (2017).

**Publisher's note:** Springer Nature remains neutral with regard to jurisdictional claims in published maps and institutional affiliations.

## Supplementary Material

Supplementary Data

## Figures and Tables

**Table 1:** Association of NF-κB p65 and survivin expression with clinicopathologic characteristics of the bladder cancer patients.

Parameters	Group	No. of cases	*P*-value	
			NF-κB p65	survivin
Gender	Male	24 (60%)	0.473	0.328
Age (years)	≥60	27 (67.5%)	0.531	0.412
Histological grade	High grade	29 (72.5%)	<0.01**	<0.01**
Muscle invasion	Positive	25 (62.5%)	<0.01**	<0.01**
Distant metastases	Positive	5 (12.5%)	0.054	<0.05*
Lymphatic invasion	Positive	7 (17.5%)	<0.05*	<0.01**

Expression of p65 and survivin was measured by IHC staining, p65 or survivin positive cell percentages per HPF were counted and statistically compared between two groups. Student’s T test was used to conduct the statistical analysis. *P < 0.05, **P < 0.01.
