# Supplementary material for: NF-κB suppresses apoptosis and promotes bladder cancer cell proliferation by upregulating survivin expression in vitro and in vivo
Source: Sci Rep. 2017 Jan 31;7:40723. doi: 10.1038/srep40723 (PMC5282527; doi:10.1038/srep40723)
Supplement: Supplementary Data [file srep40723-s1.pdf]

NF-κB suppresses apoptosis and promotes bladder cancer cell proliferation by upregulating survivin expression in vitro and in vivo

Authors and affiliations
Xiaolu Cui[1], Dezhi Shen[1], Zhe Zhang[1], Yu Zeng[1], Xuyong Lin[2], Xiankui Liu[1]*

1 Department of Urology, The First Affiliated Hospital of China Medical University, Shenyang 110001, Liaoning, China.
2 Department of Pathology, The First Affiliated Hospital and College of Basic Medical Sciences, China Medical University, Shenyang 110001, Liaoning, China.

*Corresponding author
Correspondence to:
Xiankui Liu, email: Liuxiankui@sina.com
Tel: +86-024-83283433

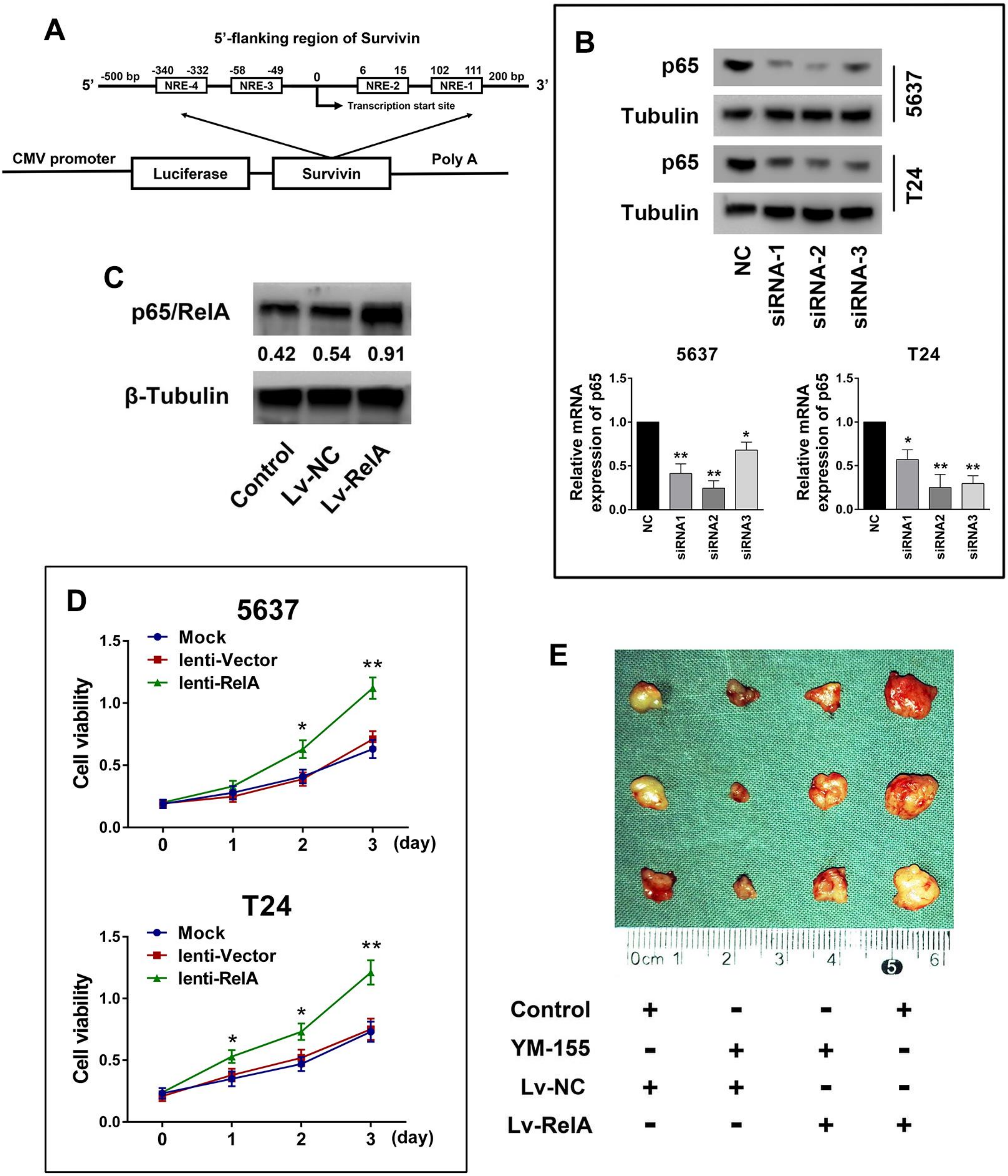
A
5'-flanking region of Survivin
5'
-500 bp
-340 -332
NRE-4
-58 -49
NRE-3
0
6 15
NRE-2
102 111
NRE-1
200 bp
3'
Transcription start site
CMV promoter
Luciferase
Survivin
Poly A
B
p65
Tubulin
p65
Tubulin
5637
T24
NC
siRNA-1
siRNA-2
siRNA-3
5637
T24
Relative mRNA expression of p65
NC
siRNA1
siRNA2
siRNA3
C
p65/RelA
0.42 0.54 0.91
β-Tubulin
Control
Lv-NC
Lv-RelA
D
5637
Mock
lenti-Vector
lenti-RelA
Cell viability
(day)
T24
Mock
lenti-Vector
lenti-RelA
Cell viability
(day)
E
0cm 1 2 3 4 5 6
Control + - - +
YM-155 - + + -
Lv-NC + + - -
Lv-RelA - - + +

Figure Legend

Figure S

(A) The 5’ flanking region of Survivin, which contains four putative NF-κB regulator elements, was cloned into a luciferase reporter vector. NRE: NF-κB regulator element. (B) Transfection efficiency of siRNA against p65 was measured by western blotting and real-time PCR analysis. β-actin was used to conduct the control for real-time PCR analysis. (C) The efficiency of lentiviral gene delivery was assessed by western blotting. The band intensities were calculated by Alpha Ease^{TM} FC software. β-Tubulin was used as an internal control. The ratio of p65 to β-Tubulin was used to conduct the statistical analysis. *$P < 0.05$ and **$P < 0.01$, as determined by Student’s T-test. (D) Cell proliferation was significantly stimulated in p65/RelA stable overexpression cells. Cell viability was determined by CCK8 assay. (E) Images of the excised tumors from each group (n = 3/group).
